# Improving the Accuracy and Training Speed of Motor Imagery Brain–Computer Interfaces Using Wavelet-Based Combined Feature Vectors and Gaussian Mixture Model-Supervectors

**DOI:** 10.3390/s17102282

**Published:** 2017-10-07

**Authors:** David Lee, Sang-Hoon Park, Sang-Goog Lee

**Affiliations:** Department of Media Engineering, Catholic University of Korea, 43-1, Yeoggok 2-dong, Wonmmi-gu, Bucheon-si, Gyeonggi-do 14662, Korea; leedabid@catholic.ac.kr (D.L.); timesea821@naver.com (S.-H.P.)

**Keywords:** brain–computer interface (BCI), electroencephalogram (EEG), training data reduction, support vector machine, motor imagery, wavelet transform

## Abstract

In this paper, we propose a set of wavelet-based combined feature vectors and a Gaussian mixture model (GMM)-supervector to enhance training speed and classification accuracy in motor imagery brain–computer interfaces. The proposed method is configured as follows: first, wavelet transforms are applied to extract the feature vectors for identification of motor imagery electroencephalography (EEG) and principal component analyses are used to reduce the dimensionality of the feature vectors and linearly combine them. Subsequently, the GMM universal background model is trained by the expectation–maximization (EM) algorithm to purify the training data and reduce its size. Finally, a purified and reduced GMM-supervector is used to train the support vector machine classifier. The performance of the proposed method was evaluated for three different motor imagery datasets in terms of accuracy, kappa, mutual information, and computation time, and compared with the state-of-the-art algorithms. The results from the study indicate that the proposed method achieves high accuracy with a small amount of training data compared with the state-of-the-art algorithms in motor imagery EEG classification.

## 1. Introduction

A brain–computer interface (BCI) refers to the technology that analyzes human’s mental activity to enable the brain to issue orders directly to computers [1,2]. BCI can help not only ordinary users, but also physically challenged persons that cannot move their muscles owing to neurological abnormalities, and the elderly and infirm, who are restricted in movement [3,4]. In general, BCIs use brain waves as input signals. However, brain waves are very sensitive to the size of the user’s head, the state of the user, the position of the electrodes, psychological conditions, the ambient environment, etc. Therefore, electroencephalography (EEG) exhibit large deviations among users. This makes BCIs trained based on a particular person difficult to use on other subjects. Therefore, BCIs require retraining whenever there are changes in the user, or in the environment or state of the user. However, retraining is quite time-consuming, and may require longer periods of time to carry out, depending upon the situation [5]. Therefore, there is a need for BCIs to improve the trade-off between accuracy and speed or strike a balance between them [6,7].

To improve BCI’s classification accuracy, recent EEG-based BCI studies have investigated and evaluated diverse feature extraction and classification algorithms [8,9,10,11,12,13]. The most common feature extraction methods include Fourier transform, wavelet transform, common spatial patterns [14,15] and auto-regressive. Classification methods include linear discriminant analysis (LDA), support vector machine (SVM), and neural network (NN). Many researchers studied motor imagery EEG signal classification using wavelet transforms, which is an effective method for multi-resolution analysis of non-stationary and transient signals, and support vector machines (SVMs) that exhibit good generalization of binary classification by maximizing the margin existing between two data classes [16,17,18,19]. Dokare et al. proposed an approach that uses wavelet coefficients and SVMs to recognize two classes of motor imagery EEG data [20]. Xu et al. used discrete wavelet transforms and fuzzy support vector machines to classify motor imagery EEG data on BCI competition II and III [21]. Eslahi et al. extracted fractal features from EEG signals and improved the accuracy of classification of imaginary hand movements using SVMs [22]. Perseh et al. improved the classification accuracy of imaginary hand movements using discrete wavelet transform (DWT) features, channel selection through Bhattacharyya distance, and SVM [23].

However, some of the BCI system studies, including the studies mentioned above, show that this method has several shortcomings. First, some motor imagery BCI studies mainly use single features. Though single features provide complementary information for identification, the combination of these single features can be considered to improve the robustness of the classifier since it has better identification power than isolated features [24,25]. Hence, combined feature vectors, rather than single features, are suitable for extracting features of non-stationary and transient signals such as EEG. Second, the standard SVMs used in BCI studies are very sensitive to the amount of training data, and to the presence of noise and outliers [26,27]. Training an SVM involves solving the convex quadratic programming (QP) (optimization) problem. When the size of the training data increases, the QP problem consumes a lot of computation time and memory. The presence of noise and outliers is inevitable in training data because it is impossible to completely avoid the programming errors and errors in the measuring equipment [28]. In particular, these problems cause very serious computational cost and accuracy issues in large training datasets [29,30]. To solve these problems, fast SVM methods such as Proximal SVM (PSVM), reduced SVM (RSVM) and Primal Estimated sub-GrAdient SOlver for SVM (PEGASOS) have been reported in literature [31,32,33]. The PSVM determines two parallel planes—each close to one of the classified data sets, while being as far as possible from each other. The RSVM randomly selects a small portion of training data to generate a thin rectangular kernel matrix. The PEGASOS solves the SVM optimization problem simply and effectively using a stochastic sub-gradient descent SVM solver. These fast SVM algorithms improve training speed but cause some loss of classification accuracy. This happens because they are based on randomly selected small training data rather than commonly used full training data. To solve these problems at once, it is necessary to maintain the distribution data of training data and to generate small training data robust to noise.

In this paper, we propose a method to improve the speed and classification accuracy of motor imagery BCI using a wavelet-based combined feature vector and a Gaussian mixture model (GMM)-supervector. The proposed method is composed of a total of four stages. First, continuous wavelet transform (CWT) and DWT are used to extract features suitable for the identification of motor imagery tasks in EEG signals. Second, principal component analysis (PCA) is used to obtain the combined feature vectors based on CWT and DWT. Third, the expectation–maximization (EM) algorithm is used to purify and transform pure training data obtained in the previous step into noise-robust small training datasets. Finally, only the mean vectors of the Gaussian mixture model universal background model (GMM-UBM) were used for the SVM training. The performance of the proposed method is evaluated using the three different BCI competition datasets (II with dataset III, III with dataset IIIb, and IV with dataset IIb) in terms of accuracy, kappa, mutual information (MI), and execution time, and compared it with state-of-the-art algorithms (PSVM, RSVM, and PEGASOS).

The remainder of this paper is organized as follows: Section 2 explains the experimental method that we propose; Section 3 presents the experiments and datasets that were used, and Section 4 presents the results of the experiments. Lastly, Section 5 presents the conclusions of this study and describes future studies.

## 2. Materials and Methods

**Figure 1:**
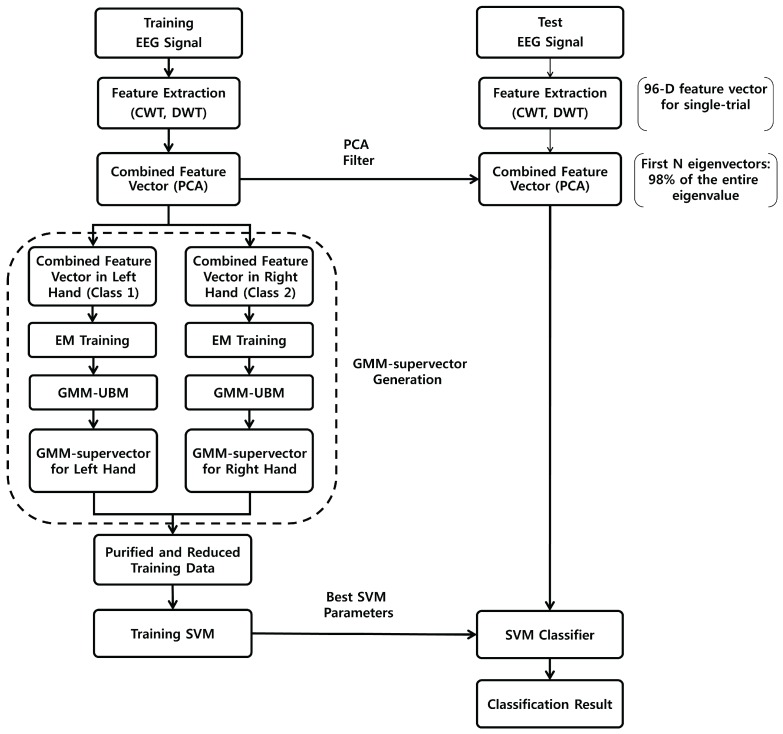
Block diagram of motor imagery brain-computer interface (BCI) using the wavelet-based combined feature vector and gaussian mixture model (GMM)-supervector.

A block diagram of the method proposed in this paper is illustrated in Figure 1. First, CWT and DWT are applied to obtain features that include both micro and macro information of the EEG signal. Then, PCA is applied to extracted features to obtain the combined feature vectors for a single trial. PCA can reduce the dimensions of the features, while maximizing orthogonal variations between features [34]. Therefore, the combined feature vectors are independent of each other, while retaining the existing information as much as possible. Next, the EM algorithm is used to optimize the pure training data of each class into noise-robust small training datasets. Finally, the mean vector of the optimized data and the reduced GMM-UBM is used to train the SVM classifier.

### 2.1. Description of the Data

To evaluate the performance of the proposed method, we used the BCI competition II [35], III [36], and IV [37], provided by the College of Biomedical Engineering, Medical Informatics of Graz University of Technology. All three datasets were obtained while the test subjects were performing left and right hand motor imagery. We only used the C3 and C4 channels that are related to the left and right hand motor imagery [38]. Table 1 shows the number of training data points and test data points for 12 subjects from BCI Competition II, III, and IV. Except for some subjects, we used all of the usable EEG signals in the motor imagery period. In order to extract features, we used the time interval between 0.5 and 6 s for BCI II, between 0.5 and 4 s for BCI III, and between 0.5 and 4.5 s for BCI IV after the cue onset. In the case of subject 1, EEG signals between 0.5 and 2 s after the cue onset were used. In the case of subject 3, EEG signals between the cue and 2 s after the cue onset were used. In the case of subject 10, EEG signals between 0.5 and 1 s after the cue onset were used and in the case of subject 12, EEG signals between 0.5 and 1.5 s after the cue onset were used.

#### 2.1.1. BCI Competition II, Dataset III

BCI competition II dataset III is a two-class EEG dataset recorded from a normal subject (25-year-old female, subject 1). This experiment consists of a total of 280 trials. The length of each trial is 9 s. EEG signals were recorded for channels C3, Cz, and C4 at a sampling rate of 128 Hz and were band-pass filtered between 0.5 Hz and 30 Hz (for details, see [35]).

#### 2.1.2. BCI Competition III, Dataset IIIb

BCI Competition III Dataset IIIb was recorded with two classes (left hand, right hand) of motor imagery EEG data from three test subjects (O3VR, S4, and X11). Of them, only data from S4 and X11 (subjects 2 and 3) were used in the present experiments because data from O3VR not only used a different paradigm, but also some trials were overlapping. The number of trials for each test subject is 1080. The length of each trial is 7 s. EEG signals were sampled at a rate of 125 Hz and were recorded using three bipolar channels (C3, C4, and Cz). Along with notch filtering, the signals were band-pass filtered between 0.5 and 30 Hz (for details, see [36]).

#### 2.1.3. BCI Competition IV, Dataset 2b

The BCI competition IV dataset 2b consisted of two classes (left hand and right hand) of motor imagery EEG data from nine test subjects (subjects 4–12). This dataset consists of five sessions and the first two sessions do not include any feedback, while the remaining sessions include feedback. Along with notch filtering (50 Hz), the EEG signals were band-pass filtered between 0.5 and 100 Hz. EEG signals were recorded using three channels (C3, Cz, and C4) at a sampling frequency of 250 Hz. In the present study, this dataset was pre-treated through a band-pass filter, between 0.5 and 30 Hz, in order to use the same frequency band as other datasets.

### 2.2. Combined Feature Vectors Based on Wavelets and PCA

While conducting motor imagery work, special features termed as event-related desynchronization (ERD) and event-related synchronization (ERS) are generated from mu waves and beta waves throughout the entire area of the sensory motor cortex [39]. In many BCI studies, such ERD/ERS components appearing from mu and beta waves were used as features for identification of EEG signals in motor imagery. However, the dominant frequency bands where ERD/ERS appear are subject-specific. Therefore, a wider frequency band is used rather than a frequency band that is of an accurate width [40]. In the present study, we use not only the 8–30 Hz band (that includes mu and beta waves), but also some theta waves, as theta waves also include EEG features for identification in motor imagery [41]. However, since EEG signals are compromised by artifacts, such as eye blinking, eye movements, and muscle noises, we excluded the bands below 6 Hz [42]. Therefore, we use the 6–30 Hz band.

Different methods such as fast Fourier transform (FFT), short-time Fourier transform (STFT), and wavelet transform (WT) can be used to analyze EEG signals. However, since EEG signals are non-stationary signals, flexible multi-resolution-based WT is more effective than FFT and STFT (which have constant frequency resolutions) for EEG analysis [8].

WT is generally categorized into CWT and DWT. To solve the problem of resolution limitation in STFT, CWT uses wavelets that are continuously adjusted and transformed. Since the wavelets used are non-orthogonal, CWT provides redundant representations of signals [43]. DWT is similar to CWT. However, to remove redundancy, DWT uses wavelets that have been discretely adjusted and changed [44]. Therefore, CWT is more appropriate if knowing the specific frequency component is important. On the other hand, DWT is more appropriate if the aggregate information in certain frequency bands is necessary [45]. In the present study, both CWT and DWT were applied to extract useful information on motor imagery (micro and macro).

#### 2.2.1. Feature Extraction by CWT

Since CWT uses windows with variable widths, it is a multi-resolution analysis method that has better frequency resolution than STFT. Let us assume that f(t) is a square integrable function. Then, the CWT of continuous-time signal f(t) provides an *A* (number of scales) × *L* (length of signals) matrix of wavelet coefficients through the following equation:

(1) $$CWT\left(a,b\right)=\int_{\infty }^{-\infty }f\left(t\right)\frac{1}{\sqrt{a}}\psi^{*}\left(\frac{t-b}{a}\right)dt,$$

where 0<a<∞ is the scale, *b* is the translation, $\frac{1}{\sqrt{a}}$ is the normalizing factor, and $\psi^{*}\left(\frac{t-b}{a}\right)$ is the complex conjugate of the mother wavelet ψ(t) in scale a and translation *b* [46,47]. CWT(a,b) indicates the degree of matching between f(t) and $\psi^{*}\left(\frac{t-b}{a}\right)$. In the present study, as mother wavelets, we used Morlet wavelets that have been used in previous BCI studies [48,49,50,51] because Morlet wavelets are appropriate for localizing frequency characteristics in time. The power of the wavelet coefficients provides the distributions of individual frequency components in time. Based on a previous study [51], the CWT feature vectors were generated by using log-transformation of power with a band width of 1 Hz between 6 and 30 Hz. The feature vector $C_{n}$ of CWT was defined as follows:

$$C_{n}=\left\{C_{n,1},C_{n,2},\ldots ,C_{n,48}\right\}.$$

Here, n=1,…,N indicates the trial index, and the respective integers indicate channel (2) × frequency band (24) = 48.

#### 2.2.2. Feature Extraction by DWT

DWT decomposes signals into coarse approximation and detailed information to analyze signals in different frequency bands with different resolutions [52]. DWT can be defined as shown below:

(2) $$DWT\left(j,k\right)=\sum_{i}\sum_{k}f\left(t\right)2^{-\frac{j}{2}}\Psi \left(2^{-j}t-k\right),$$

where f(t) represents functions of time *t*. Ψ represents wavelet basis functions and *j* and *k* represent frequency resolution and travel time for the time axis. EEG signals were decomposed into four or five levels using Daubechies wavelet 4 (db4). D2, D3, and D4 (or D3, D4, and D5) include the beta, mu, and theta waves, respectively, where the features (ERD/ERS) of motor imagery EEG appear. Therefore, these sub-bands have been used to classify EEG signals. The following statistics of extracted wavelet coefficients were used as features [52,53]:

(1) Mean of the absolute values of the wavelet coefficients in each sub-band
(2) Average power of the wavelet coefficients in each sub-band
(3) Standard deviation of the wavelet coefficients in each sub-band
(4) Ratio of the absolute mean values of adjacent sub-bands
(5) Energy of the wavelet coefficients in each sub-band
(6) Entropy of the wavelet coefficients in each sub-band
(7) Skewness of the wavelet coefficients in each sub-band
(8) Kurtosis of the wavelet coefficients in each sub-band

Features 1 and 2 indicate frequency distributions, features 3 and 4 indicate the variance in frequency distributions, and features 5 and 6 indicate time-frequency distributions [54]. Feature 7 indicates the measurements of the asymmetry of frequency distribution, and feature 8 indicates the measurements of the peaks of frequency distributions [55]. The feature vector computed for mu, beta and theta waves for EEG signal classification were defined as follows:

$$D_{n}=\left\{D_{n,1},D_{n,2},\ldots ,D_{n,48}\right\}.$$

Here, the respective integers indicate channel (2) × frequency band (3) × statistic (8) = 48.

#### 2.2.3. Combined Feature Vectors by PCA

As explained above, CWT was applied to extract data every 1 Hz bin width within the 6–30 Hz band. DWT was applied to extract theta, mu, and beta waves associated with motor imagery. In the present study, the features extracted in this manner are combined to form a single feature vector. This method gives macro and micro information in time and frequency, enhancing the accuracy of motor imagery classification. To form the composite feature vectors, we connected all of the feature vectors of CWT and DWT. The composite feature vector consists of a total of 96 features:

$$F_{n}=\left\{C_{n,1},\ldots ,C_{n,48},D_{n,1},\ldots ,D_{n,48}\right\}.$$

In general, the dimensions of a composite feature vector are large. For small training samples, because the amount of training data may be less than the number of features, classification algorithms such as SVMs suffer from the curse of dimensionality [56]. Therefore, in order to prevent such a problem, it is crucial to reduce the dimensionality of the features. Among dimensionality reduction methods, PCA has been widely used in diverse areas [57,58]. The purpose of PCA is to find the linear orthogonal transformation matrix (projection matrix *W*) that has the maximum variance in a dataset consisting of many variables, while reducing the dimensions of the dataset [59]. The PCA projection matrix *W* can be obtained by selecting *K* eigenvectors that have the highest eigenvalues. We use the first *K* components that contain 98% of variance in the data. Therefore, the reduced combined feature vectors $S\in {ℜ}^{N\times K}$ can be obtained by applying the PCA projection matrix *W* to our composite feature vectors set *F* as shown in the following equation:

(3) S=FW.

### 2.3. GMM-Supervectors

SVMs have high computational cost due to large amounts of data required for the training process, which is sensitive to noise. One way to solve these problems is to reduce the size of the training dataset [60]. The proposed method uses the GMM to generate a noise-robust small training dataset from the original training data. Subsequently, the obtained mean vector of the Gaussian components was used as the new training dataset for the SVM. The new small training dataset contains the information of the original training data, while also reducing the size. Here, we applied the concept of the method proposed in [61] to generate GMM-supervectors.

The GMM is a generative model to estimate clustering or densities using statistical methods. Since it also uses covariance matrices, it includes information about the distribution of features, and has generative models that are relatively less impacted by noise than discriminative models. Using the GMM method, the overall training data can be expressed through a sufficiently small number of representative training samples. In GMM, the distribution of training data for each class is modeled using $m_{l}$ Gaussian components. Therefore, the GMM is a parametric probability density function expressed as the sum of the weights of Gaussian densities of the components [62]. Suppose we have the GMM-UBM using the EM algorithm to generate representative training samples. The GMM-UBM is defined as:

(4) $$p\left(s_{l}|\theta_{l}\right)=\sum_{i=1}^{m_{l}}\omega_{l,i}g\left(s_{l}\mu_{l,i},\Sigma_{l,i}\right).$$

$s_{l}$ are the *K*-dimensions feature vectors for class l(l={1,2}), and $\theta_{l}=\left\{\omega_{l,i},\mu_{l,i},\Sigma_{l,i}\right\}$ is a set of all parameters of the GMM-UBM in class *l*. $\omega_{l,i}$, $\mu_{l,i}$ and $\Sigma_{l,i}$ are the prior probabilities (mixture weight), average vector, and diagonal covariance matrix for the *i*th element of class *l*. Here, $\omega_{l,i}$ is a probability value and therefore must satisfy $0\leq \omega_{i}\leq 1$, $\Sigma_{i=1}^{m_{l}}\omega_{l,i}=1$. $g\left(s_{l}|\mu_{l,i},\Sigma_{l,i}\right)$ shows the Gaussian component of the *i*th element for class *l*. In a *K*-dimensional space, the respective Gaussian components were defined as follows:

(5) $$g\left(s_{l}|\mu_{l,i},\Sigma_{l,i}\right)=\frac{1}{{\left(2\pi \right)}^{K/2}{\left|\left|\Sigma_{l,i}\right|\right|}^{1/2}}\exp\left\{-\frac{1}{2}{\left(s_{l}-\mu_{l,i}\right)}^{T}\Sigma_{l,i}^{-1}\left(s_{l}-\mu_{l,i}\right)\right\}.$$

Given $m_{l}$ components, the complete set $\theta_{l}$ of GMM-UBM parameters was determined by maximum likelihood [63]. For class 1, the data was used by the EM algorithm to generate the parameters of the GMM-UBM. Only the mean vectors from the GMM-UBM were selected, which are referred to as the GMM-supervectors [61]. The GMM-supervectors for class 2 are obtained using the same procedure. Subsequently, in order to create a representative training sample that is robust against noise, we connected the GMM-supervectors for each class. As a result, together with the selected mean vectors, we are able to form the following representative training sample, which represents the overall training data:

$$\begin{array}{c} \left[\begin{array}{c} \mu_{1,1}\;\;\cdots \;\;\mu_{1,k}\\ \vdots \;\;\;\;\ddots \;\;\;\vdots \\ \mu_{m_{1},1}\;\cdots \;\;\mu_{m_{1},k}\\ \mu_{m_{1}+1,1}\;\;\cdots \;\;\mu_{m_{1}+1,k}\\ \vdots \;\;\;\ddots \;\;\;\vdots \\ \mu_{m_{1}+m_{2},1}\;\;\cdots \;\;\mu_{m_{1}+m_{2},k} \end{array}\right]. \end{array}$$

**Figure 2:**
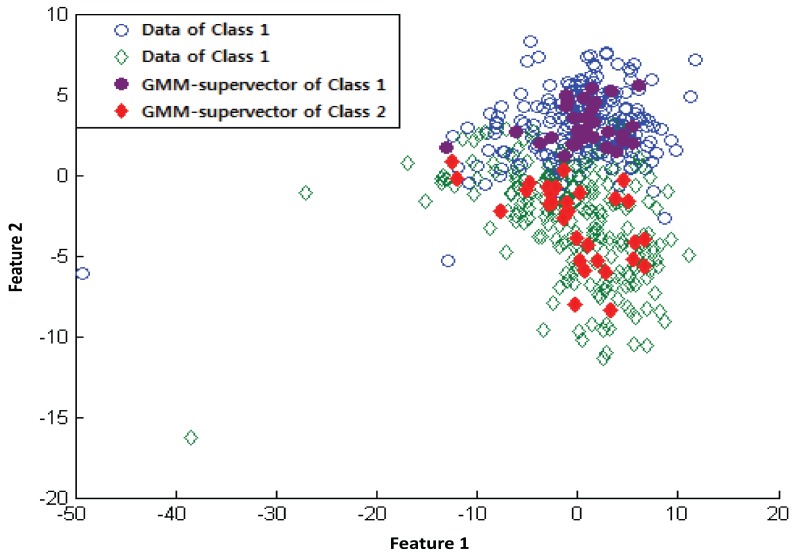
GMM-supervector for two features for Subject 7 in dataset IIb of the BCI competition IV.

Here, $\mu_{i,j}$ represents the *j*th feature of the *i*th representative training sample. $i=1,\ldots ,m_{1}$ are representative training samples belonging to class 1 and $i=m_{1}+1,\ldots ,m_{2}$ are representative training samples belonging to class 2. This representative training sample M is used as input data for the SVM. Figure 2 shows the mean vector of the class-specific GMM-supervectors generated by using two features for subject 7 in the BCI competition IV dataset IIb. The colored-in shape indicates the mean of the Gaussian components.

### 2.4. Support Vector Machine (SVM)

The SVM is a discriminative model that has been widely used for linear and nonlinear classifications due to its high prediction accuracy. The SVM determines the optimal hyperplane (i.e., decision boundary) that has the largest margin between two classes. Data from classes closest to a decision boundary are called support vectors.

When a dataset $D={\left\{x_{i},y_{i}\right\}}_{i=1}^{N}$ in which $y_{i}\in \left\{-1,1\right\}$ is given, the SVM aims to find an optimal hyperplane that separates the samples in the feature space. For a nonlinear problem, this hyperplane is given by $f\left(x\right)=\omega \cdot \Phi \left(x_{i}\right)+b$, where ω is the optimal set of weights, $\Phi (x_{i})$ represents a nonlinear mapping on $x_{i}$, and *b* is the optimal bias. The primal optimization problem of the SVM is as follows:

(6) $$\begin{array}{c} \min_{\omega ,b,\xi }\frac{1}{2}\omega^{T}\omega +C\sum_{i=1}^{N}\xi i,\\ Subject\;to\;y_{i}\left(\omega^{T}\Phi \left(x_{i}\right)+b\right)\geq 1-\xi_{i},\;\xi_{i}\geq 0, \end{array}$$

where C>0 is the penalty parameter and $\xi_{i}$ represents the degree of misclassification of the *i*th sample. This optimization problem can be solved by transforming it into a dual problem using Lagrange optimization:

(7) $$\begin{array}{c} \max\sum_{i=1}^{N}\alpha_{i}-\frac{1}{2}\sum_{i=1}^{N}\sum_{j=1}^{N}\alpha_{i}\alpha_{j}y_{i}y_{j}K\left(x_{i},x_{j}\right),\\ Subject\;to:\sum_{i=1}^{N}\alpha_{i}y_{i}=0,\;0\leq \alpha_{i}\leq C, \end{array}$$

where $\alpha_{i}$ is the Lagrange multiplier obtained by solving the quadratic programming problem. $K(x_{i},x_{j})$ is a kernel function. In the present study, radial basis kernel functions were used as the SVM’s kernel functions:

(8) $$K\left(x_{i},x_{j}\right)=\exp\left(-\frac{{\left|\left|x_{i}-x_{j}\right|\right|}^{2}}{2\sigma }\right),$$

where σ is a kernel parameter that is related to kernel width. In the present study, cross-validation (CV) was applied to evaluate and select the optimal values of *C* and σ through the grid search [64]. The range of parameters was given as follows: $C=2^{-5},\ldots ,2^{15},$$\sigma =2^{-15},\ldots ,2^{3}$. The fold number CV was set to 10.

## 3. Experimental Results

To assess the applicability of the proposed method, we conducted experiments on three different datasets recorded by the Graz BCI group (Graz University of Technology, Graz, Austria). First, by comparing the DWT, CWT, and combined feature vectors for the three datasets in terms of accuracy, we proved the efficiency of combined feature vectors. Next, to assess the efficiency of our method, we carried out a comparative experiment against state-of-the-art algorithms. The best parameters for SVM in the classification process were selected by applying 10-fold cross validation to the training data. The training data that is downsized using the proposed method is generated differently each time. To ensure more objective comparative test results, we repeated the test 10 times for each subject. In other words, 10×10-fold cross-validation was used to evaluate the performance of proposed method. The experiment was carried out using a CPU 3.4 GHz processor, 4 GB RAM, Windows 7 (Microsoft, Redmond, WA, USA), and Matlab 2014b (version 8.4, MathWorks, Natick, MA, USA).

### 3.1. Performance of the Combined Features Vector

First, we checked whether combined features are valid as features for motor imagery EEG signal classification. Table 2 shows the classification accuracy of the SVM classifier, comparing single feature extraction methods and combined feature vectors. The results show that the accuracy based on combined feature vectors with PCA are approximately 0.5 to 6.1% better than other features on average. In particular, the combined feature vectors with PCA revealed a statistically significant improvement in classification accuracy compared to DWT (*p* < 0.05) and CWT (*p* < 0.05). Although accuracy was reduced compared to CWT for Subject 4, improved accuracy was observed for the other subjects. For Subjects 8 and 10, the classification accuracy was markedly improved. The combined feature vectors using PCA show lower dimensionality and higher average accuracy compared to when PCA is not applied. Although combined feature vector with PCA showed reduction in accuracy for some subjects, on average, the accuracy was improved by approximately 0.5%. In addition, the number of features was reduced to approximately 35.7% (34.3) of the entire number of features (96).

Table 3, Table 4, and Table 5 show the performance comparison of the combined feature vectors with winning methods for each dataset, in terms of MI, accuracy, and kappa. Because three different datasets were used, the method of assessment used for comparison differs depending on the dataset. The proposed method exhibited better performance in all aspects when compared with the results for the winning methods of each dataset.

We compared the MI of the proposed method against the MI of the competition-winning methods of BCI competitions II and III. Table 3 and Table 4 show the MIs for the proposed method using all the training data for BCI competition II and III, the proposed method using 30% of all training data, and each of the BCI competition-winning methods. In Table 3, the maximum MIs for the proposed method using all and 30% of the training data were 0.84 and 0.67, respectively. These results represent 0.23 and 0.06 improvements, respectively, over the result achieved by the first winner of BCI competition II. On the other hand, in BCI competition III shown in Table 4, the proposed method using all training data, named ALL-SVM, exhibited lower performance than the first winner. However, for Subject 3, ALL-SVM had a maximum MI of 0.3562, which represents better performance than the 0.3489 of the first winner. In addition, the ALL-SVM and 30%-SVM methods both showed better performance than the second winner.

Next, we consider the kappa values of the proposed method and compare them to the winning methods of BCI competition, which has the largest number of subjects (Table 5). The results show that the ALL-SVM method had a better mean kappa value (0.62) than the first winner (0.60). The results also show that the ALL-SVM method was better than the first winner for five subjects (4, 5, 8, 9, and 10). It was shown that, while 30%-SVM achieved similar kappa values to the second winner, the kappa values were less than those of the first winner in most of the subjects. However, in some subjects (4 and 9), the kappa result was quite high.

### 3.2. Performance of a Fast and Robust SVM Training Method

**Figure 3:**
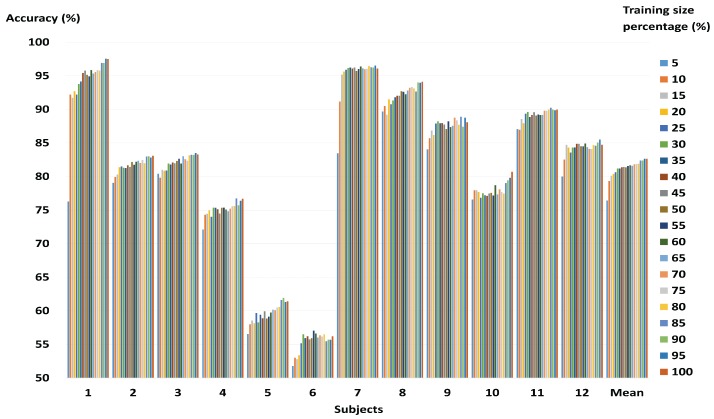
Classification accuracy based on reduction rate of whole training data on individual subjects.

In this subsection, we evaluate classification accuracies depending on the size m of the representative training sample, and experimental results depending on training time. Figure 3 shows that, as the size of the training dataset increases, the change in accuracy according to reduction rate remains constant. For subjects 9 and 11, when the size of the training data was reduced to 15%, and 25%, lower losses (less than 1%) were observed. For subjects 2 and 3, when the size of the training data was reduced to 65%, losses of 1% were observed. On the other hand, for subject 1, because the initial training dataset was small (140), the difference in accuracy according to reduction rate was greater than for the other subjects.

**Figure 4:**
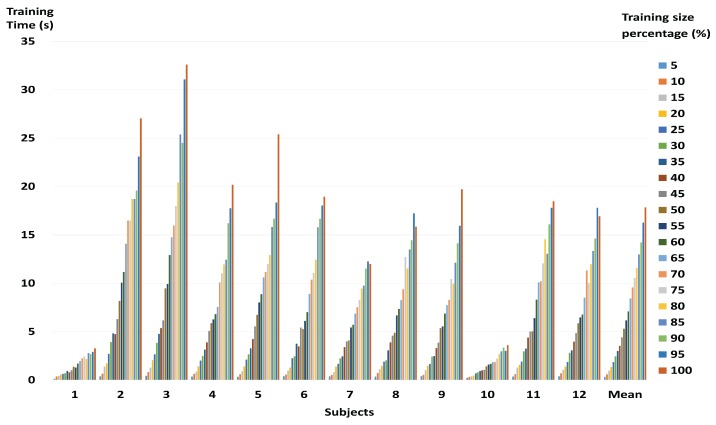
Computation time for training procedure based on reduction rate of whole training data on individual subjects.

The proposed method shows average losses of 1.47% in classification accuracy when the training dataset has been reduced to 30%. However, as can be seen in Figure 4, the mean speed of the training process for all subjects increased approximately 7.30 times on average. Looking at subjects with small training datasets only, the improvement was 1.18 times on average, while for the rest of the subjects with large training datasets, the average improvement was 7.36. In particular, the speed of the training process for subject 9 was observed to increase by up to 14.35 times with a loss of some 1% when the size of the training dataset was reduced to 15%.

### 3.3. Comparison with State-of-the-Art Algorithms

To compare our method against current SVM methods for BCI systems, we carried out comparative experiments against state-of-the-art algorithms such as PSVM, PEGASOS, and RSVM, which are effective in enhancing SVM speed. Library for SVM (LIBSVM), which uses random sampling, was used as another reference algorithm for performance assessment (also called Random method). This is the most widely used SVM algorithm. In the experiments, the state-of-the-art algorithms used wavelet-based combined feature vectors as the input. The proposed method used the GMM-supervector generated by wavelet-based combined feature vector as the input. For each of the three datasets, 10×10-fold cross-validation was used to evaluate the performance of the classifiers. The proposed method and the state-of-the-art SVM algorithms were trained to classify the motor imagery EEG.

**Figure 5:**
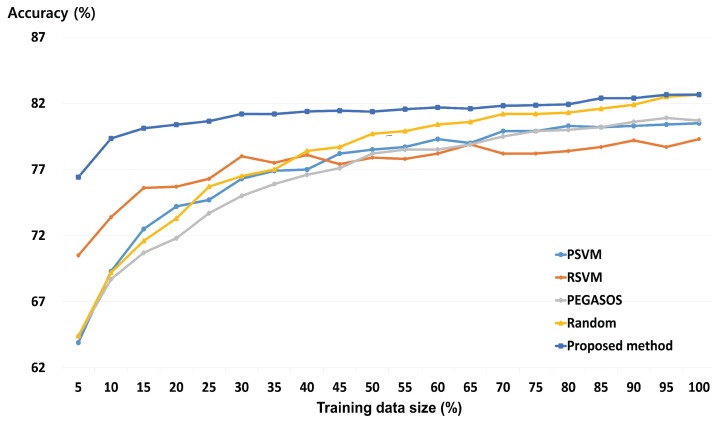
Mean classification accuracy based on selected training data on all subjects.

Figure 5 shows the mean classification accuracies of the proposed method and four state-of-the-art algorithms depending on the size of the training dataset for all subjects. It is clearly shown that the proposed method achieves higher accuracies than all of the other state-of-the-art algorithms at all reduction rates. The greater the reduction rate of the training data, the greater the difference in classification accuracy between the proposed method and other state-of-the-art algorithms. While the proposed method has an accuracy of 76.4% when using only 5% of training data, the accuracies for PSVM, RSVM, PEGASOS, and Random method were 63.9%, 70.5%, 64.4%, and 64.4%, respectively. Comparing the cases where all training data is used and where 30% of the training data is used, the proposed method has a classification accuracy loss of 1.47% on average. On the other hand, the classification accuracy losses for PSVM, RSVM, PEGASOS and Random method when compared to 100% training data use were 2.0%, 1.4%, 2.5%, and 3.1%, respectively. When compared against the highest classification accuracies, the classification accuracy losses for PSVM, RSVM, and PEGASOS were 4.2%, 4.8%, and 4.5%, respectively. These differences increase with the reduction rate.

**Figure 6:**
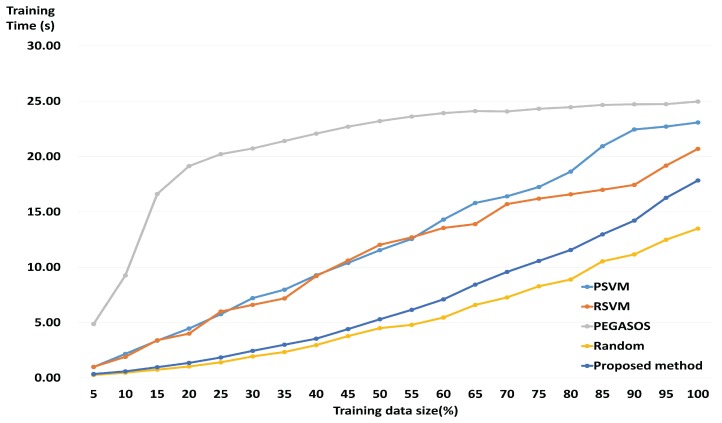
Computation time for training procedure based on selected training data on all subjects.

Figure 6 shows the mean training time of the proposed method and the state-of-the-art algorithms depending on the size of the training dataset for all subjects. Training time of the proposed method includes the GMM-supervector generation process. Nevertheless, the proposed method becomes faster than PSVM, RSVM and PEGASOS. Comparing the cases where all training data is used and where 30% of the training data is used, the proposed method was almost 7.30 times faster. On the other hand, PSVM, RSVM, PEGASOS and Random method when compared to using the entire training data, increased almost 3.71 times, 3.14 times, 1.20 times, and 6.96 times, respectively. In other words, the proposed method showed the greatest decrease in training time.

## 4. Discussion

In the present study, we used wavelet-based combined feature vectors as features suitable for classification of motor imagery EEG signals. As shown in Table 2, it was confirmed that, while the classification accuracy gained using DWT was lower than that for CWT, DWT itself had information based on which motor imagery EEG signals could be classified. That is to say that DWT, which contains macro information relating to a broad frequency band, and CWT, which contains micro information relating to a narrow 1 Hz band, have potential as features of motor imagery BCI. The data in DWT represents macro information, including information on a broad frequency band, and the data in CWT represents micro information on a narrow 1 Hz band. As macro data is a trend created by a collection of micro data, it can be said to be the effect of a cause. Having both cause and effect is more helpful in resolving problems than having only the cause or only the effect. This was ultimately proven by the combined feature vectors (shown in Table 2) exhibiting higher classification accuracy than individual features, in most of the subjects. Figure 3 shows that, for some subjects, the classification accuracy for the representative training sample is higher than when all the training data was used. Therefore, it may be said that the proposed method helps to improve the classification accuracy of the existing SVM. Furthermore, the representative training sample generated through GMM, which is robust against over-fitting, is more helpful in improving the generalization ability of the SVM than the existing training data. Figure 3 also shows that, if the amount of training data is large, the loss in accuracy is not significant, even at higher reduction rates. This means that the proposed method is more useful in reducing large training datasets than small training datasets. In BCI applications, both accuracy and speed are important. Therefore, it is important to reduce the size of the training data while maintaining as high a level of accuracy as possible. The proposed method was shown to have similar accuracy as state-of-the-art algorithms at both high and low reduction rates. These results indicate that the proposed method, by evaluating an appropriate representative training sample for the overall training data, helps to maintain classification accuracy. The results also show that, as the proposed method has achieved good classification accuracy using less training data than state-of-the-art algorithms, it is able to carry out a faster training process. That is, the comparative experiment against state-of-the-art algorithms proves that the proposed method is effective and stable. In addition, when using only 30% of the training data, the training time of the proposed method is about 2.3 s. This value is faster than other fast SVMs except the Random method. Therefore, the proposed method does not significantly affect computation time for the training process. Test time is short (about 0.004 s) in each training data size. Therefore, the proposed method can be applied to real-time BCI system implementation.

## 5. Conclusions

A wavelet and PCA-based combined feature vectors method and the fast and robust SVM training method have been proposed to improve the accuracy and speed of motor imagery BCI. The proposed method reduced the overall training data size to 30% while achieving low loss (1%). Moreover, the speed of SVM training was increased by up to a factor of 7.30. In addition, because of the smaller number of training data points, testing speed was naturally improved. The proposed combined feature vectors methods were superior to methods with a single feature for most subjects. Although the combined feature vectors exhibited lower classification performance than single features in some subjects, the differences observed were at acceptable levels. The GMM-based fast and robust SVM training method showed better trade-offs between accuracy and speed than the state-of-the-art algorithms. The method proposed in the experiment, by exhibiting high reduction rates for large training data alongside good classification accuracy, showed high potential for use in BCI applications. On the other hand, while the proposed method exhibited good classification accuracy for small training datasets, it exhibited relatively low reduction rates for large training datasets. While the proposed method is effective, there are some remaining issues to be resolved. The first is that the times at which EEGs associated with motor imagery occur vary from person to person and trial to trial. For this reason, in the experiment conducted in this study, we used different motor imagery periods for some subjects. However, this choice was empirical. Therefore, future studies will focus on developing methods to automatically set optimal motor imagery periods for subjects. The second is the question of what the optimal reduction rate of training data is. The reduction rate varies depending on the size of the training data. With a loss of 1% as the standard, our results showed a high reduction rate for a large amount of training data, while the reduction rate for a small amount of training data was low. Accordingly, we will consider methods of automatically setting the optimum reduction rate while maintaining accuracy and reducing the SVM training data size as much as possible. Lastly, it was shown that the combined feature vectors and training data reduction method was effective in motor imagery EEG signals with two classes. Accordingly, by applying the method to motor imagery EEG signals with more classes, we will investigate the versatility of this method. Fourth, we will consider common spatial pattern (CSP), one of the widely used feature extraction methods, to extract features that are more suitable for motor imagery-based BCI. CSP has been used in many studies related to motor imagery. Ang et al. found motor imagery for control and rehabilitation by improving the signal-to-noise ratio of EEG using CSP [12]. Zhang et al. optimized the spatial pattern of EEG using the modified CSP [14]. We will therefore consider a new combined feature extraction method that combines CSP and time-frequency methods in future work. Fifth, the proposed GMM-supervector method reduces the size of large training samples to speed up the training of the SVM. In other words, the proposed methods and traditional fast SVMs take a different approach, but they have the same purpose: to speed up the training. However, since the proposed method reduces the training sample, it can be combined with other classifiers such as LDA, quadratic discriminant analysis (QDA), and logistic classification as well as SVM. There are some exceptions, however, in some optimization methods using a random sampling or subset of training sample, and the proposed method may not be suitable because training data reduction is applied twice. Therefore, we will investigate the combined scalability of GMM-supervector method and other classifiers in future work. Lastly, we will consider a multimodal signal [66] that combines EEG with additional psychophysiological signals such as the pupil, and deep learning to further improve classification accuracy of motor imagery-based BCIs.

## Figures and Tables

**Table 1:** Summary information of the data for 12 subjects from BCI Competition II, III, and IV.

Subject	1	2	3	4	5	6	7	8	9	10	11	12
**Number of Training Data Points**	140	540	540	400	400	400	420	420	400	400	440	400
**Number of Test Data Points**	140	540	540	320	280	320	320	320	320	320	320	320

**Table 2:** Comparative results of the feature extraction methods in terms of the average classification accuracy (%).

Subject	DWT	CWT	Combined Feature Vectors			
			without PCA		with PCA	
			Accuracy	Number of Features	Accuracy	Number of Features
**1**	92.9	94.1	96.4	96	97.5	33
**2**	73.3	81.4	83.1	96	83.1	40
**3**	62.5	80.7	80.5	96	83.3	39
**4**	75.2	77.7	79.1	96	76.7	35
**5**	52.6	60.1	60.0	96	61.4	34
**6**	55.4	55.6	51.8	96	56.2	32
**7**	95.5	96.2	96.2	96	96.1	30
**8**	88.3	87.4	94.0	96	94.1	32
**9**	79.2	87.8	90.2	96	88.1	34
**10**	74.8	72.6	81.4	96	80.7	36
**11**	90.6	87.7	89.3	96	90.0	32
**12**	78.7	83.3	84.2	96	84.8	34
**Mean**	76.6	80.4	82.2	96	82.7	34.3
***p*-value**	*p* < 0.05	*p* < 0.05	*p* = 0.20	-	-	-

DWT: discrete wavelet transform, CWT: continuous wavelet transform, PCA: principal component analysis.

**Table 3:** Mutual information of the proposed combined feature vectors (100% and 30% of training data), methods in previous literature [21,65], and the winning methods of dataset I of BCI Competition II.

Ranking	Methods	Subject 1	
		Maximal MI (bit)	Accuracy (%)
**1**	ALL-SVM	0.84	97.50
**2**	30%-SVM	0.67	93.79
**3**	FSVM in [21]	0.66	87.86
**4**	SVM in [21]	0.65	89.83
**5**	NN in [65]	0.64	90.00
**6**	LDA in [65]	0.63	89.29
**7**	1st winner	0.61	89.29
**8**	SVM in [65]	0.58	90.00
**9**	2nd winner	0.46	84.29

SVM: support vector machine, FSVM fuzzy SVM, NN: neural network, LDA: linear discriminant analysis.

**Table 4:** Mutual information of the proposed combined feature vectors (100% and 30% of training data), methods in [21] and the winning methods of dataset II.

Ranking	Methods	Maximal MI(bit)		
		Subject 2	Subject 3	Mean
**1**	1st winner	0.4382	0.3489	0.3936
**2**	ALL-SVM	0.3447	0.3562	0.3505
**3**	30%-SVM	0.3105	0.3216	0.3161
**4**	2nd winner	0.4174	0.1719	0.2947
**5**	FSVM in [21]	0.0718	0.0863	0.0791
**6**	SVM in [21]	0.0718	0.0809	0.0764

**Table 5:** Maximum Kappa value of the proposed combined feature vectors (100% and 30% of training data), and winning methods of the dataset III.

Ranking	Methods	4	5	6	7	8	9	10	11	12	Mean
**1**	ALL-SVM	0.54	0.24	0.12	0.92	0.88	0.76	0.61	0.80	0.70	0.62
**2**	1st winner	0.40	0.21	0.22	0.95	0.86	0.61	0.56	0.85	0.74	0.60
**3**	30%-SVM	0.51	0.17	0.12	0.92	0.83	0.76	0.55	0.79	0.67	0.59
**3**	2nd winner	0.42	0.21	0.14	0.94	0.71	0.62	0.61	0.84	0.78	0.59
**5**	3rd winner	0.19	0.12	0.12	0.77	0.57	0.49	0.37	0.85	0.61	0.45

## Abbreviations

The following abbreviations are used in this manuscript:

BCI	Brain–Computer Interface
CV	Cross-Validation
CWT	Continuous Wavelet Transform
CSP	Common spatial pattern
DWT	Discrete Wavelet Transform
EEG	Electroencephalography
EM	Expectation–Maximization
ERD	Event-Related Desynchronization
ERS	Event-Related Synchronization
FFT	Fast Fourier Transform
GMM	Gaussian Mixture Model
GMM-UBM	Gaussian Mixture Model Universal Background Model
LDA	Linear Discriminant Analysis
PCA	Principal Component Analysis
STFT	Short-Time Fourier Transform
SVMs	Support Vector Machines
WT	Wavelet Transform
